# Atopy is a risk factor for respiratory symptoms in COPD patients: results from the EUROSCOP study

**DOI:** 10.1186/1465-9921-14-10

**Published:** 2013-01-28

**Authors:** Fatemeh Fattahi, Nick H T ten Hacken, Claes-Göran Löfdahl, Machteld N Hylkema, Wim Timens, Dirkje S Postma, Judith M Vonk

**Affiliations:** 1 Department of Pulmonology, University of Groningen, University Medical Center Groningen, PO Box 196 9700 AD, Groningen, The Netherlands; 2 Department of Pathology and Medical Biology, University of Groningen, University Medical Center Groningen, Groningen, The Netherlands; 3 Groningen Research Institute for Asthma and COPD (GRIAC), University of Groningen, University Medical Center Groningen, Groningen, The Netherlands; 4 Department of Respiratory Medicine and Allergology, University Hospital, Lund, Sweden; 5 Department of Epidemiology, University of Groningen, University Medical Center Groningen, Groningen, The Netherlands

**Keywords:** Allergy, Chronic obstructive pulmonary disease, Corticosteroid, Gender, Lung function, Respiratory symptoms

## Abstract

**Background:**

The pathogenesis of COPD is complex and remains poorly understood. The European Respiratory Society Study on Chronic Obstructive Pulmonary Disease (EUROSCOP) investigated long-term effects of budesonide; 18% of the COPD participants were atopic. So far effects of atopy on the long-term course of COPD have not been elucidated.

**Methods:**

Factors related to the presence of atopy (positive phadiatop) in 1277 mild-to-moderate COPD patients participating in EUROSCOP were analysed using regression analysis. Incidence and remission of respiratory symptoms during 3-year follow-up were analysed using generalised estimating equations models, and association of atopy with lung function decline using linear mixed effects models.

**Results:**

Independent predisposing factors associated with the presence of atopy were: male gender (OR: 2.21; 95% CI: 1.47–3.34), overweight/obese (OR: 1.41; 95% CI: 1.04–1.92) and lower age (OR: 0.98; 95% CI: 0.96–0.99). Atopy was associated with a higher prevalence of cough (OR: 1.71; 95% CI: 1.26–2.34) and phlegm (OR: 1.50; 95% CI: 1.10–2.03), but not with lung function levels or FEV_1_ decline. Atopic COPD patients not treated with budesonide had an increased incidence of cough over time (OR: 1.79, 95% CI: 1.03–3.08, p = 0.038), while those treated with budesonide had increased remission of cough (OR: 1.93, 95% CI: 1.11–3.37, p = 0.02) compared to non-atopic COPD patients.

**Conclusions:**

Atopic COPD patients are more likely male, have overweight/obesity and are younger as compared with non-atopic COPD patients. Atopy in COPD is associated with an increased incidence and prevalence of respiratory symptoms. If atopic COPD patients are treated with budesonide, they more often show remission of symptoms compared to non-atopic COPD patients who are treated with budesonide. We recommend including atopy in the diagnostic work-up and management of COPD.

## Background

Atopy, coming from the Greek *atopos*, meaning “out of place”, refers to the hereditary predisposition to produce Immunoglobulin E (IgE) antibodies against common environmental allergens. This may lead to clinical expression of atopic diseases such as allergic rhinitis, asthma and atopic eczema [1]. The prevalence of atopic disorders has increased over recent decades [2] and a decrease in microbial exposure and changes in lifestyles (e.g. dietary habits, obesity, less physical activity) have been suggested to be causative factors [3].

In asthma, most patients have an atopic phenotype [1], a feature that is associated with less severe disease and better lung function [4]. Furthermore, it is well known that atopic asthmatics respond better to corticosteroids as they have an eosinophilic inflammatory pattern in the airway wall compared to non-atopic asthmatics [5,6]. Therefore, recommendations for the treatment of allergy are also included in the treatment guidelines of asthma. In contrast, in chronic obstructive pulmonary disease (COPD), a common and disabling smoking-related disease responsible for considerable morbidity and mortality worldwide [7], the international diagnostic and treatment guidelines do not incorporate recommendation for the treatment of allergy. This lack of recommendations is largely due to insufficient knowledge on the role of atopy in the pathogenesis and outcome of COPD. Nevertheless, it has been reported that around 18% of COPD patients are atopic [8,9] and that atopy is a possible risk factor for developing COPD [10-13]. Therefore, there has been a growing interest in finding the link between atopy and COPD and its consequence on the disease outcome. However, the effect of atopy on respiratory symptoms or lung function in COPD patients has not been studied yet. Understanding this issue is of clinical importance as it may help to know the prognosis and to apply appropriate medical interventions for atopic and non-atopic COPD patients.

The European Respiratory Society Study on Chronic Obstructive Pulmonary Disease (EUROSCOP) has measured the atopic status in its study population. EUROSCOP is a large multi-centre study performed in 39 centers in 9 European countries and has been designed to assess the effect of 3-year treatment with inhaled budesonide on lung function decline in smoking COPD patients. The results showed a small improvement in lung function after 6 months in the treated group but no differences in long-term lung function decline [8]. So far, the effect of atopy on lung function and respiratory symptoms was not evaluated in this large longitudinal study. Therefore, we assessed factors associated with the presence of atopy in this COPD population and investigated whether there is a difference between atopic and non-atopic COPD patients regarding prevalence, incidence and remission of respiratory symptoms as well as lung function decline over the 3-year follow-up of the study.

## Methods

### Subjects

We analysed data from the EUROSCOP study [8,9,14], included 1277 COPD patients (from nine European countries) aged 30–65 years who had failed to quit smoking during a 3-month smoking-cessation program. They were currently smoking ≥ 5 cigarettes/day, had smoked for ≥10 years or had a smoking history of ≥5 packyears. Post-bronchodilator FEV_1_ was between 50 and 100% of the predicted value, and the ratio of pre-bronchodilator FEV_1_ to slow vital capacity (VC) was <70%. Subjects with a history of asthma, reversible airflow limitation, any atopic diseases like allergic rhinitis, or allergic eczema and those who had used oral glucocorticoids for ≥4 weeks during the prior six months were excluded. The participants were allocated to two treatment groups in a randomised, double-blind parallel-controlled way, either receiving twice daily 400 μg budesonide (Pulmicort, Astra, Stockholm, Sweden) or placebo from a dry-powder inhaler (Turbuhaler, Astra) for a period of 3 years. Approval from regulatory and ethics committees was obtained at all centers. All subjects gave written informed consent.

### Measurements

At baseline height, weight, and smoking habits were assessed. BMI (weight/height^2^) was divided into 3 categories: underweight (<18.5 kg/m^2^), normal weight (18.5–24.9 kg/m^2^), and overweight/obesity (≥25 kg/m^2^) [15]. Atopy was determined in 1163 patients by measuring specific IgE, using the Phadiatop test (Pharmacia & Upjohn, Uppsala, Sweden). Total serum IgE level was measured in 678 patients [8]. Information on respiratory symptoms was assessed at baseline and annually thereafter [8]. The symptoms analysed in the present study were: 1) cough in the morning, during the day or at night in winter, 2) phlegm in the morning, during the day or at night in winter, 3) wheezing/whistling in the chest at any time, 4) attacks of shortness of breath after activity, 5) ever trouble with breathing and 6) woken with a feeling of tightness in the chest [9].

Spirometry, using the criteria of the American Thoracic Society [16], was performed at baseline and at 3-monthly intervals using a dry rolling-seal spirometer. Post-bronchodilator FEV_1_ was obtained 15 min after inhalation of 1 mg terbutaline [8]. Reference values of the European Respiratory Society [17] were used to calculate FEV_1_% predicted.

### Statistical analyses

1) Possible predictors for the presence of atopy (positive Phadiatop) were analyzed using univariate analyses at baseline. Given the fact that the prevalence of atopy differs between males and females [18-20], we stratified for gender. Differences between atopics and non-atopics were assessed in the gender strata using 2-sample Student’s *t* test or rank-sum test (where appropriate) for continuous variables (age and packyears) and *χ*^2^ test or Fisher’s exact test for categorical variables (sex and BMI). Subsequently a multivariate (unstratified) model adjusted for gender was performed using logistic regression, including all variables with a p value <0.30 in either the male or female univariate analysis. As the number of subjects with underweight was very low (Table 1), we combined underweight with normal weight in the multivariate regression analyses.

**Table 1:** Characteristics of atopic and non-atopic COPD patients in the EUROSCOP study stratified by gender

**Baseline variables**	**Males (843 patients)**			**Females (320 patients)**		
	**Atopic**	**Non-atopic**	**p. value**	**Atopic**	**Non-atopic**	**p. value**
	**n = 181**	**n = 662**		**n = 32**	**n = 288**	
**Age, yr**	53.0 (48.0–58.0)	54.0 (48.0–59.0)	0.164	51.0 (46.0–58.7)	52.0 (47.0–58.0)	0.391
**Height, cm**	176 (172–181)	176 (171–180)	0.403	165.5 (160.5–169.7)	165 (160–169)	0.494
**Weight, kg**	80.0 (72.0–88.0)	78.0 (70.0–85.0)	**0.017**	64.0 (58.5–73.7)	62.0 (55.0–70.0)	0.206
**BMI, kg/m**^**2**^	25.3 (23.5–27.5)	24.8 (22.6–27.1)	**0.016**	23.8 (21.8–25.9)	22.9 (21.0–25.4)	0.205
**Underweight (<18.5)**	2 (1.1%)	7 (1.1%)		0 (0.0%)	17 (5.9%)	
**Normal weight (18.5–24.9)**	77 (42.5%)	342 (51.7%)	0.102^#^	20 (62.5%)	186 (64.6%)	0.277^#^
**Overweight/Obese (≥25)**	101 (55.8%)	312 (47.1%)		12 (37.5%)	84 (29.2%)	
**Packyears of smoking**	40.0 (29.2–55.5)	38.7 (28.5–50.0)	0.288	32.5 (26.7–36.0)	29.9 (21.3–39.0)	0.462
**FEV**_**1**_, liter^*^	2.7 (2.4–3.3)	2.8 (2.3–3.2)	0.680	2.1 (1.6–2.4)	2.0 (1.7–2.4)	0.904
**FEV**_**1 **_% pred.^*^	78.7 (69.4–87.0)	79.3 (68.1–89.1)	0.685	79.4 (63.6–89.2)	80.8 (70.5–88.5)	0.666
**FEV**_**1**_%FVC^*^	63.9 (56.7–68.5)	64.4 (58.1–68.7)	0.443	66.4 (61.4–70.0)	65.5 (60.9–70.4)	0.823
**Reversibility % pred.**	2.9 (0.8–5.2)	2.8 (0.0–5.4)	0.932	3.2 (0.3–7.6)	2.9 (0.0–5.5)	0.323
Total IgE, kU/l^**^	248.5 (84.0–617.2)	37.0 (15.0–82.0)	**<0.0001**	161.0 (25.5–1373.0)	28.0 (13.0–75.0)	**0.002**

Data are presented as median (interquartile range) or number (%). Bold p-values lower than 0.05 indicate significant differences between atopic and non-atopic patients within males or females.

*Respiratory function tests were performed after inhalation of 1 mg terbutaline.

**Available in 678 patients.

^#^P.value refers to Chi-square analysis between classes of BMI and atopy.

2) Differences in the prevalence of respiratory symptoms at baseline between atopics and non-atopics were analyzed using *χ*^2^ test. Multiple logistic regression adjusted for sex, age, BMI and packyears was performed for each symptom separately to investigate the association between atopy and respiratory symptoms at baseline. This analysis was performed for the total population, stratified for gender, and interactions between atopy and gender were investigated.

3) Association between atopy and changes in the presence of respiratory symptoms during the study period were analysed using generalised estimating equation (GEE) models as described by Watson et al. [9]. In brief, pairs of observations were formed between the baseline and the first 12-monthly visit (0–12 months), and between 12–24 months and 24–36 months. In the analysis on symptom incidence, only paired observations where the symptom under study was not present at the first observation of the pair were included. The symptom status at the second observation of the pair was taken as the outcome variable. For the analyses on symptom remission only paired observations where the symptom under study was present at the first observation of the pair were included. Each person could contribute one to three paired observations. For the incidence and remission of each symptom, Odds ratios (ORs) for atopy were calculated. These analyses were performed for the two treatment groups separately as ICS treatment may modify the association between atopy and symptom incidence/remission. In addition, this effect modification by treatment was investigated by entering an interaction term between atopy and treatment in the unstratified models. The analyses were further stratified by gender and were adjusted for age, BMI, atopy, packyears, and FEV_1_ % predicted, all measured at baseline.

4) To investigate the association between atopy and FEV_1_ decline over time, linear mixed effects models were used. Since the FEV_1_ decline in EUROSCOP is not linear over time, two separate periods (0–6 and 6–36 months) were investigated [8]. The models were stratified for gender and treatment group and adjusted for age, packyears and height.

## Results

### Baseline characteristics

The baseline characteristics of the atopic and non-atopic males and females are shown in Table 1. In total, 213 (18.3%) patients were atopic. Atopy was more prevalent in males than females [21.5% and 10% respectively, p < 0.001]. Atopic males had a higher weight (p = 0.017) and BMI (p = 0.016) than non-atopic males. Atopic patients had significantly higher total serum IgE (kU/l) levels than non-atopic patients (p < 0.0001 in males and p = 0.002 in females). There were no significant differences in age and lung function parameters at baseline between atopic and non-atopic patients.

### Factors associated with the presence of atopy

Multiple logistic regression analysis showed that male gender (OR: 2.21; 95% CI: 1.47–3.34), overweight/obese (OR: 1.41; 95% CI: 1.04–1.92) and lower age (OR: 0.98; 95% CI: 0.96–0.99) were independently associated with the presence of atopy. There was no significant association between the number of packyears (OR: 1.007; 95% CI: 0.99–1.01) and the presence of atopy.

### Atopy and respiratory symptoms

Atopic patients had a higher prevalence of cough (p = 0.02) and phlegm (p = 0.08) than non-atopic patients (Table 2). After stratifying by gender, a higher prevalence of cough (p < 0.0001) and phlegm (p = 0.008) was found in atopic males than non-atopic males, without a significant difference in females (Table 2). Woken with chest tightness was more prevalent in atopic females than non-atopic females (p = 0.042) (Table 2). In the multiple logistic regression model adjusted for confounders, atopy was associated with a higher prevalence of cough (OR: 1.71; 95% CI: 1.26–2.34) and phlegm production (OR: 1.50; 95% CI: 1.10–2.03) in the total population, and with woken with chest tightness in females only (OR females: 2.69; 95% CI: 1.11–6.55, OR male: 0.84; 95% CI: 0.47–1.49, female vs male: OR: 3.21; 95% CI: 1.12–9.25).

**Table 2:** Respiratory symptoms in atopic and non-atopic COPD patients at baseline stratified by gender

**Respiratory symptoms**	**Total population**		**Males**		**Females**	
	**1163 patients**		**843 patients**		**320 cases**	
	**Atopic**	**Non-atopic**	**Atopic**	**Non-atopic**	**Atopic**	**Non-atopic**
	**n (%)**	**n (%)**	**n (%)**	**n (%)**	**n (%)**	**n (%)**
**Subjects number**	213 (18.3)	950 (81.7)	181 (21.5)	662 (78.5)	32 (10)	288 (90)
**Wheezing at anytime**	111 (52.1)	527 (55.5)	95 (52.5)	353 (53.3)	16 (50)	174 (60.4)
**Cough day/night or a.m.**	155 (72.8)*	609 (64.1)	110 (60.8)*	291 (44.0)	18 (56.2)	170 (59.0)
**Phlegm day/night or a.m.**	127 (59.6)^#^	498 (52.4)	87 (48.1)*	244 (36.9)	12 (37.5)	101 (35.1)
**Trouble with breathing**	104 (48.8)	443 (46.7)	85 (47.0)	287 (43.4)	19 (59.4)	156 (54.2)
**Woken with chest tightness**	24 (11.3)	102 (10.7)	16 (8.8)	68 (10.3)	8 (25.0)*	34 (11.8)
**Attack of dyspnea after activity**	82 (38.5)	350 (36.8)	68 (48.1)	234 (35.3)	14 (43.8)	116 (40.3)

*Significant difference between atopic and non-atopic patients at p < 0.05. ^#^: p = 0.08.

### Atopy and incidence and remission of symptoms

The association between atopy and incidence and remission of symptoms during the 3 years of the study, stratified by treatment group and gender is shown in Tables 3 and 4 respectively.

**Table 3:** Association between atopy and incidence of respiratory symptoms stratified by the treatment group and gender

**Respiratory symptom**	**Total population**		**Males**		**Females**	
	**OR (95% CI) of atopy**		**OR (95% CI) of atopy**		**OR (95% CI) of atopy**	
	**Placebo**	**Budesonide**	**Placebo**	**Budesonide**	**Placebo**	**Budesonide**
**Wheezing at any time**	1.15 (0.71–1.87)	0.79 (0.45–1.43)	1.24 (0.74–2.08)	0.92 (0.48–1.75)	0.64 (0.15–2.78)	0.43 (0.10–1.85)
**Cough day/night or a.m.**	1.79 (1.03–3.08)^*^	0.83 (0.42–1.62)	1.69 (0.93–3.08)^#^	0.84 (0.38–1.84)	2.52 (0.64–9.86)	0.60 (0.17–2.08)
**Phlegm day/night or a.m.**	1.50 (0.84–2.69)	0.91 (0.54–1.53)	1.55 (0.82–2.93)	0.85 (0.46–1.59)	1.30 (0.36–4.67)	0.92 (0.38–2.27)
**Trouble with breathing**	1.12 (0.66–1.89)	0.68 (0.37–1.24)	1.09 (0.59–1.99)	0.79 (0.42–1.51)	1.23 (0.44–3.44)	0.22 (0.4–1.13)^#^
**Woken with chest tightness**	1.33 (0.74–2.38)	1.07 (0.57–1.99)	1.35 (0.69–2.65)	1.08 (0.55–2.12)	1.08 (0.27–4.34)	1.11 (0.23–5.39)
**Attack of dyspnea after activity**	0.98 (0.59–1.62)	1.49 (0.88–2.51)	0.76 (0.43–1.33)	1.55 (0.88–2.73)^$^	2.79 (0.94–8.31)^#^	0.89 (0.22–3.62)

*OR is significant at p < 0.05. Each model was adjusted for sex, age, BMI, pack years of smoking, number of cigarettes and FEV_1_ % pred.

#Trend (0.05 < p. value < 0.1).

$Interaction between Phadiatop and treatment group has a p.value < 0.05.

**Table 4:** Association between atopy and remission of respiratory symptoms classified by the treatment group and gender

**Respiratory symptom**	**Total population**		**Males**		**Females**	
	**OR (95% CI) of phadiatop**		**OR (95% CI) of phadiatop**		**OR (95% CI) of phadiatop**	
	**Placebo**	**Budesonide**	**Placebo**	**Budesonide**	**Placebo**	**Budesonide**
**Wheezing at any time**	0.99 (0.59–1.67)	1.18 (0.70–1.99)	0.88 (0.48–1.60)	1.26 (0.70–2.26)	2.06 (0.55–7.78)	0.51 (0.15–1.71)
**Cough day/night or in a.m.**	0.85 (0.53–1.36)	**1.93 (1.11–3.37**)^*,$^	0.87 (0.52–1.45)	1.94 (1.05–3.57)^*^	0.79 (0.26–2.41)	1.84 (0.47–7.30)
**Phlegm in day/night or in a.m.**	1.21 (0.72–2.03)	1.67 (0.99–2.82)^#^	1.14 (0.65–2.00)	1.63 (0.93–2.87)^#^	1.48 (0.34–6.45)	1.53 (0.28–8.26)
**Trouble with breathing**	0.84 (0.48–1.47)	1.76 (0.99–3.11)^#^	0.96 (0.50–1.82)	2.76 (1.45–5.26)^*,$^	0.47 (0.18–1.21)	0.43 (0.17–1.12)^#^
**Woken with chest tightness**	2.18 (0.97–4.90)^#^	2.32 (0.60–8.91)	2.17 (0.67–6.99)	1.33 (0.22–7.85)	5.76 (1.67–19.86)^*^	8.82 (0.63–123.66)
**Attack of dyspnea after activity**	1.01 (0.52–1.95)	1.11 (0.63–1.96)	1.04 (0.49–2.20)	0.87 (0.46–1.64)	1.03 (0.29–3.63)	3.67 (0.68–19.70)

*OR is significant at p < 0.05. Each model was adjusted for sex, age, BMI, pack years of smoking, number of cigarette and FEV_1_ % pred.

#Trend (0.05 < p. value < 0.1). $Interaction between Phadiatop and treatment group has a p.value < 0.05.

In the placebo group, atopy was significantly associated with an increased incidence of cough (OR: 1.79, 95% CI: 1.03–3.08, p = 0.038). Atopy was not significantly associated with the incidence of the other symptoms.

Analyses on remission of symptoms showed that remission of cough was higher in atopic than non-atopic patients receiving budesonide (OR: 1.93, 95% CI: 1.11–3.37, p = 0.02). After stratifying by gender, remission of cough was higher in atopic males than non-atopic males receiving budesonide (OR: 1.94, 95% CI: 1.05–3.57, p = 0.034) as was trouble with breathing (OR: 2.76, 95% CI: 1.45–5.26, p = 0.002), but differences were not present in female subjects receiving budesonide. In contrast, atopic females receiving placebo, had increased remission of woken with chest tightness (OR: 5.76, 95% CI: 1.67–19.86, p = 0.006) than non-atopic females receiving budesonide.

**Figure 1:**
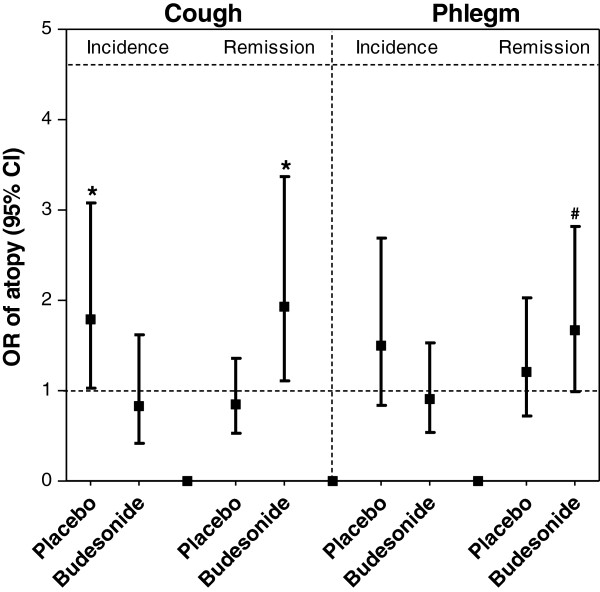
**Effect of atopy on incidence and remission of cough and phlegm in the treatment groups.** Logistic regression with adjustment for age, BMI, packyears, and FEV_1_ % predicted. * p < 0.05, # p = 0.056.

The incidence and remission of cough and phlegm production in two treatment groups are shown in Figure 1.

### Atopy and lung function decline

**Figure 2:**
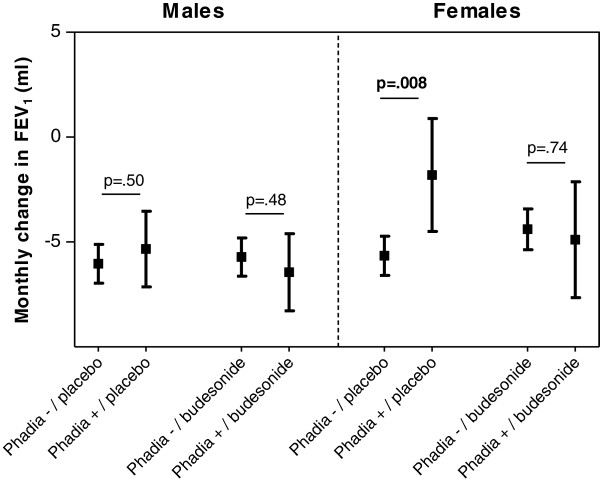
**Monthly change of FEV**_**1 **_**from 6 months to 36 months after the start of the study medication.** Linear mixed effect models with adjustment for age, BMI, atopy, packyears, and FEV_1_ % predicted.

There was no significant difference in changes of post-bronchodilator FEV_1_ between atopic and non-atopic patients neither in males nor in females, a finding that was true for both placebo and budesonide treated groups during month 0 to 6 (Figure 2). From 6 to 36 months, atopic females who received placebo showed a smaller decline in FEV_1_ compared to the non-atopic females in the placebo group (p = 0.008, Figure 2).

## Discussion

Our study shows that male gender, overweight/obesity and lower age are independently associated with the presence of atopy in COPD. Moreover, atopic patients showed a higher prevalence of respiratory symptoms than non-atopics. Interestingly, atopic patients without ICS treatment more frequently developed respiratory symptoms than non-atopics, while atopic patients on treatment with ICS showed increased remission of respiratory symptoms compared to non-atopic patients.

We found that the prevalence of atopy is two times higher in males than females both in univariate and multivariate analyses. This confirms previous findings in the literature. Sears et al. found that boys (age of 13 years) had a higher prevalence of positive skin tests and a higher response to house dust mite and cat than girls with the same age [21]. With increasing age, a significant decrease in male/female ratio of sensitization was described after the age of 8 years although a male predominance persists [18] also in older men [19,20]. This can be explained by a population study in adults which showed that atopy significantly decreased after menopause in both asthmatic and non asthmatic women, suggesting that the pathophysiology of atopy changes over the lifespan depends on the hormonal pattern [19]. We corroborate these findings by showing a male preponderance of atopy in COPD.

Younger age was also associated with the presence of atopy in our COPD patients. This finding is in line with results from studies in the general population showing that allergen sensitivity and the incidence of atopic disorders decreases with age [22-25].

Another interesting finding in our study was that overweight/obesity was associated with the presence of atopy in COPD patients. The previous studies in the general population also showed a significant association between overweight/obesity and atopy in adolescents [26] and in adults [24]. In asthma, it has been suggested that the systemic inflammatory effects of obesity itself may enhance eosinophilic airway inflammation [27]. We do not know whether this is also true for COPD and the atopy-overweight/obesity relationship in COPD has to be further explored.

With respect to respiratory symptoms, our study revealed a higher prevalence of cough and phlegm in atopic COPD patients compared to non-atopic COPD patients indicating that atopy (i.e. positive phadiatop) contributes importantly to symptoms in COPD. The association between atopy and a higher prevalence of respiratory symptoms was also found in the general population, as various respiratory symptoms have been associated with positive skin test reactivity [10,28] and eosinophilia [28,29]. But in COPD, according to our knowledge, there is no published paper showing an association between atopy and respiratory outcomes. One recent ATS abstract [30] is in line with our findings, investigating 1424 COPD patients from “The National Health and Nutrition Examination Survey (NHANES)” III (1988–1994). The investigators defined allergic/atopic COPD subjects (n = 346) as the presence of any one of the following criteria: at least one positive skin prick test, self-reported doctor diagnosed hay fever, or symptoms induced by house dust, animals or pollen. They found that individuals with indications of allergic disease more likely reported having episodes of sinusitis, and an additional trend towards more frequent reporting of cough and wheeze [30] compared to non-allergic individuals. Our study defined atopy objectively by specific IgE positivity and *excluded* subjects with a history of asthma, allergic rhinitis, or allergic eczema. As we excluded subjects with allergic diseases, we believe our data more closely reflects the effect of atopy on COPD-related cough. Regarding the importance of cough and phlegm, it should be noted that these symptoms are highly prevalent in COPD patients and have been reported to predict disease progression, exacerbations and hospitalizations [31]. It has been argued that these symptoms can constitute a sign of inflammation and may identify patients at higher risk of clinical worsening [31]. Thus, our finding that atopy associates with this clinical phenotype may have important consequences for future studies on intervention in this phenotype with an important clinical impact on COPD, as shown in our study.

Our study did not show a significant difference in lung function parameters between atopic and non-atopic patients, with the exception of FEV_1_ decline. Of interest, atopic female COPD patients not using ICS treatment demonstrated a slower decline in lung function than non-atopic females. Additionally, if atopic females used ICS this protective effect of atopy was no longer present. In established COPD, to our knowledge, such an effect of atopy has never been investigated. We do not have a clear explanation for the latter finding, but as this observation is not present in male subjects, we speculate that hormonal-related effects on the immune system play a role. However, the number of atopic females in our study was low (n = 32); so firm conclusions cannot be drawn.

It has been shown that atopy is associated with a lower level of lung function [32,33] and FEV_1_ decline [34] in the general population and also FEV_1_ decline in healthy former and current smokers [35]. We conclude that, unlike in healthy subjects, atopy is not associated with accelerated decline in lung function in established COPD. It may well be that the effects of atopy are overshadowed by the effects of smoking in our COPD population.

Our study showed that in atopic COPD patients the use of budesonide is associated with higher remission rates of cough and phlegm, whereas placebo is associated with higher incidence rates. This is an important finding as cough and phlegm predict disease progression, exacerbations and hospitalizations [31]. Although this beneficial effect of budesonide may not be specific for atopic COPD and may be present in every atopic subject, the question rises whether we should treat **all atopic** COPD patients with an ICS (as EUROSCOP included only steroid-naïve patients). Indeed, already in 1978, Sahn suggested that atopic COPD patients are the ones who benefit most from corticosteroid treatment [36]. If we accept that atopic COPD patients from now on should be treated with ICS, this would widen the present indications for ICS as defined by GOLD (Global Initiative for Chronic Obstructive Lung Disease) [37]. At this moment GOLD recommends ICS use for symptomatic patients with an FEV_1_ < 50% predicted (stage III, severe COPD, and stage IV, very severe COPD) and repeated exacerbations [37,38]. However, before considering to add atopic status as a guideline for ICS treatment in COPD, more studies are needed confirming that atopy is a risk factor for worse COPD outcome.

## Conclusion

We conclude that atopy is present in COPD patients and that the prevalence of atopy is higher in males, subjects with overweight/obesity and younger patients. Importantly, atopy in COPD is associated with a higher prevalence and incidence of respiratory symptoms, while when being treated with ICS, the patients have higher remission rates. However, atopy in COPD is not associated with accelerated but rather decelerated FEV_1_ decline in females. Our results clearly indicate that the atopic status should not be forgotten in the routine work-up of COPD. However, whether every atopic COPD patient should be treated with an ICS needs to be confirmed in future studies.

## Competing interests

The authors declare that they have no competing interests.

## Authors’ contributions

JMV had full access to all of the data in the study and takes responsibility for the integrity of the data and the accuracy of the data analysis and contributed to the revision of the manuscript. FF: contributed to the data analysis, interpreting of the results and manuscript writing. NHTtH: contributed to the data analysis, interpreting of the results and manuscript writing. C-GL: contributed to the original study design and conception, acquisition of the data, and revision of the manuscript. MNH and WT: contributed to interpreting of the results and revision of the manuscript. DSP: contributed to the original study design and conception, acquisition of the data, and revision of the manuscript. Other contributions: The data analysed in this study were collected in the EUROSCOP-study. We thank all principle investigators and participants of the EUROSCOP study. All authors read and approved the final manuscript.
